# Regular and irregular stimuli result in changes in mice eye movement and cerebellar nuclei neuron model behavior

**DOI:** 10.1186/1471-2202-16-S1-P202

**Published:** 2015-12-18

**Authors:** Tiina Manninen, TD Barbara Nguyen-Vu, Jennifer L Raymond

**Affiliations:** 1 Department of Signal Processing, Tampere University of Technology, Tampere, Finland; 2 Department of Neurobiology, Stanford School of Medicine, Stanford, CA 94305, USA

## 

The cerebellum plays an important role in motor control. The cerebellar key players include Purkinje cells (PCs), mossy fibers, climbing fibers, and parallel fibers. Each PC receives inputs from many parallel fibers and from a single climbing fiber. Cerebellar outputs originate from the deep cerebellar and vestibular nuclei. Cerebellar nuclei (CN) neurons receive inhibitory inputs from PCs and excitatory inputs from mossy fiber and climbing fiber collaterals. In this work, we studied how regular and irregular, as well as synchronous and asynchronous, PC firing frequencies affect the eye movements in mice and CN neuron model behavior. Floccular PCs on one side of the head were optogenetically stimulated (see, e.g., [1]). We used both regular and irregular Poisson distributed stimulus trains of 10-90 Hz. As the experimental output, we measured the horizontal eye movements. We compared the effects of the parameters of the stimulus train in the actual eye movements elicited with the effects of the same parameters on the computational CN neuron model. The CN neuron model [2,3] includes 517 compartments and it receives an inhibitory input from 450 PC synapses originating from 1-450 individual PCs and an excitatory input from 150 mossy fiber synapses. The model has eight different types of ion channels represented with Hodgkin-Huxley type equations. We used both regular and Poisson distributed PC and gamma distributed mossy fiber spike trains as inputs. We ran the model in NEURON simulation environment [4] and did all the data analysis in MATLAB®. We varied the input irregularity, synchrony, mean firing rate (20-120Hz), and PC to CN neuron convergence (1-450). As the model output, we measured the cerebellar nuclei neuron firing rate and mean GABA conductance. The experiments and the model simulations exhibited similar behavior for some but not all of the stimulus frequencies. This suggests a need for further experimental and simulation studies to more fully understand how the parameters of PC firing rate influence downstream oculomotor circuits.
